# Nanocomposite Hydrogels with Polymer Grafted Silica Nanoparticles, Using Glucose Oxidase

**DOI:** 10.3390/gels9060486

**Published:** 2023-06-13

**Authors:** Ali A. Mohammed, Siwei Li, Tian Sang, Julian R. Jones, Alessandra Pinna

**Affiliations:** 1 Dyson School of Design Engineering, Imperial College London, London SW7 9EG, UK; ali.mohammed@imperial.ac.uk; 2 Department of Materials, Imperial College London, London SW7 2AZ, UK; siwei.li@vssacademy.co.uk (S.L.); tsang1535@hotmail.com (T.S.); julian.r.jones@imperial.ac.uk (J.R.J.); 3 The Francis Crick Institute, London NW1 1AT, UK; 4 School of Veterinary Medicine, Faculty of Health and Medical Sciences, University of Surrey, London GU2 7XH, UK

**Keywords:** polymer grafted silica nanoparticles, redox polymerisation, biomaterials, glucose oxidase, hydrogels, tissue engineer

## Abstract

Nanocomposite hydrogels offer remarkable potential for applications in bone tissue engineering. They are synthesized through the chemical or physical crosslinking of polymers and nanomaterials, allowing for the enhancement of their behaviour by modifying the properties and compositions of the nanomaterials involved. However, their mechanical properties require further enhancement to meet the demands of bone tissue engineering. Here, we present an approach to improve the mechanical properties of nanocomposite hydrogels by incorporating polymer grafted silica nanoparticles into a double network inspired hydrogel (gSNP Gels). The gSNP Gels were synthesised via a graft polymerization process using a redox initiator. gSNP Gels were formed by grafting 2-acrylamido-2-methylpropanesulfonic acid (AMPS) as the first network gel followed by a sequential second network acrylamide (AAm) onto amine functionalized silica nanoparticles (ASNPs). We utilized glucose oxidase (GOx) to create an oxygen-free atmosphere during polymerization, resulting in higher polymer conversion compared to argon degassing. The gSNP Gels showed excellent compressive strengths of 13.9 ± 5.5 MPa, a strain of 69.6 ± 6.4%, and a water content of 63.4% ± 1.8. The synthesis technique demonstrates a promising approach to enhance the mechanical properties of hydrogels, which can have significant implications for bone tissue engineering and other soft tissue applications.

## 1. Introduction

Hydrogels are hydrophilic three-dimensional polymeric networks that hold large amounts of water (up to 99%), giving them their intrinsically soft material properties. They hold numerous properties including flexibility, transparency, permeability, biocompatibility, and low friction [1,2,3]. Hydrogels can be formed through natural, synthetic, and hybrid polymers, offering a wide range of biochemical and mechanical properties [4,5]. Single network hydrogels are usually soft, weak, and brittle. Their applications are limited due to their tensile and compressive properties being in the sub-MPa range, and inability to withstand strains greater than 100% compared with the hydrogel-like natural-tissues such as articular cartilage, muscle, tendon, and blood vessels [6].

Hydrogel technology has evolved beyond its initial single network systems, driven by the inherent physical and biochemical limitations they possess [7]. This progress has led to the emergence of several innovative and practical hydrogel subtypes, including interpenetrating network hydrogels (IPNs), nanocomposite hydrogels, stimuli-responsive hydrogels, and double network hydrogels (DNHGs). DNHGs consist of two contrasting polymeric networks of polyelectrolytes (rigid and brittle) and neutral polymers (soft and ductile). The opposing properties of both networks have potential for achieving synergy of the mechanical properties of the two networks, replicating the physical and biochemical properties of native human tissue such as articular cartilage and bone [8,9,10]. This improvement in mechanical properties is crucial for bone tissue engineering, where different bone types exhibit varying compressive strengths. For instance, cortical bone demonstrates an ultimate compressive strength ranging from 130 to 180 MPa, while trabecular bone typically has a compressive strength of 1 to 5 MPa [11].

DNHGs are normally synthesized using a two-step sequential free-radical polymerisation (FRP) where a high relative molecular weight neutral second polymer network is swollen within a lightly cross linked heterogeneous first network polyelectrolyte [10]. DNHGs have been developed using a wide range of pairs consisting of polyelectrolyte first networks and neutral second networks, which have shown enhanced mechanical properties when compared to their individual network hydrogels [9]. A DNHG developed by Gong [10] consisting of poly(2-acrylamido 2-methylpropanesulfonic acid)/polyacrylamide (PAMPS/PAAm) had a compressive fracture stress of up to 20 MPa at 92% strain whilst holding 90 wt.% water. These properties closely matched that of native articular cartilage and trabecular bone [10]. In contrast, the respective single network hydrogel components had sub-MPa fracture stresses at 80% strain for PAAm and 40% for PAMPS. However, upon bearing stress, the covalent bonds in conventional DNHGs exhibit permanent damage; therefore, the gels cannot recover from large deformations and they suffer low fatigue resistance [9,12,13]. To improve on these limitations, additives such as nanoparticles can be introduced to create microstructures that increase resistance to strain and increase stiffness.

The introduction of nanocomposite structures such as silica nanoparticles (SNPs) [14,15], copper nanopowder [16,17], laponite clay [17], superparamagnetic iron oxide nanoparticles (SPIONs) [18], nanoceria (NC) [19], and nanocellulose crystals (CNC) [20,21,22] have shown to further enhance the physical properties of hydrogels. Nanocomposite hydrogels can provide unique properties based on the type of nanoparticle introduced into the system. Properties can include internal physical reinforcement and stiffness [8,14,19], topography for cellular anchorage crucial for cell adhesion and tissue regeneration [23], conductivity [24], antibacterial, antioxidation [25,26,27], therapeutic ion release [28,29], cancer therapeutics [30], magnetic responsiveness [18,31], and electrical signals and sensing. Improvements in the properties of DNHGs through the addition of nanocomposite structures have also been reported, e.g., mechanical improvements through the addition of graphene oxide in alginate/nanofibrillated cellulose [32], and development of thermoresponsive properties using as poly(*N*-isopropylacrylamide) (PNIPAAm) with polysiloxane nanoparticles [33]. This expands their potential applications in specialized tissues such as skeletal muscle, nerve, and cardiac tissues.

However, nanocomposite gel structures are challenging to produce due to particle agglomeration, inhomogeneous distribution during synthesis, and a lack of covalent or ionic bonds between particles and the polymer networks [34,35,36,37,38]. This could result in weaknesses and potential points for failure when the hydrogel is under mechanical stress. To avoid these issues and to ensure a more robust entanglement of polymer chains with the nanocomposites, polymers can be grafted directly onto the nanoparticles to form strong covalent bonds. In previous studies, polymers were successfully grafted on the surface of SNPs, for example: polymethyl methacrylate (PMMA) by graft photopolymerisation [39], polystyrene with living radical polymerisation [40], and with atom transfer radical polymerisation (ATRP) [41]; poly-n-isopropylacrylamide (PNIPAM) brushes by ATRP [42], polystyrene sulfonic acid sodium salt (PSSA), and PAMPS by surface initiated redox polymerisation [43]. In a previous study, PAMPS and PSSA were grafted on the surface of amine-functionalized SNP (ASNPs) [43]. The ASNPs were dispersed in acidic solution of AMPS monomers before both the initiator ceric ammonium nitrate (CAN) and stabilizer sodium dodecyl sulphate (SDS) were added. The mixture was degassed with nitrogen gas and heated to 40 °C to initiate the graft polymerisation. Grafting was initiated by a redox pair of Ce (IV) (oxidant) and the alkyl amine (reductant) from the surface of the ASNPs, causing an intermediate free radical at the α-carbon atom of the alkyl amine group. However, graft percentage and polymer conversion were low, at 46% and 4.6% for PAMPS, and 22% and 2.2% for PSSA, respectively. These low percentages may cause issues in hydrogel formation due to low polymer grafts and extra post processing steps to remove unreacted monomers. The study aimed at increasing polymer graft percentage; however, attempts at hydrogel synthesis were not made. An optimization of the process would be required to create a polymer-nanocomposite that has the potential to be used in the synthesis of polymer grafted silica nanoparticle gels (gSNP Gels). In our previous work, polymers were successfully grafted on the surface of cerium oxide using a novel auto-catalytic graft polymerisation to form DNHGs for articular cartilage repair [19]. The process used a self-initiating cyclical polymerisation technique that benefited from the two alternating cerium ion states (Ce^3+^ and Ce^4+^) and reaction by-product H_2_O_2_.

In this work, a method for synthesizing a double network inspired nanocomposite hydrogel is presented. The method involves using a redox initiator, ceric ammonium nitrate, in combination with glucose oxidase (GOx), an oxidoreductase enzyme that can quench oxygen in an open system, eliminating all oxygen inhibition steps that impact FRP and increase the conversion of the polymer [14]. The redox polymerization is used to graft polymers with high conversion on ASNPs, and GOx is used to enhance the kinetic profile of each polymer by degassing all saturated oxygen in the monomer solution, allowing for a more efficient graft polymerization process to take place. To demonstrate the efficacy of GOx, a comparison with argon degassing will be made for both AMPS and AAm. The gSNP Gels are produced by grafting AMPS as a first network polyelectrolyte on the surface of ASNPs, followed by a sequential graft polymerization of AAm on ASNPs as the second network. Both networks are synthesized in oxygen-free atmospheres in the presence of GOx. The goal of this approach is to achieve improved integration of the nanocomposite structure through a polymer grafting process compared to conventional mixing of nanoparticles during synthesis.

## 2. Results and Discussion

### 2.1. Amine Functionalized Silica Nanoparticles (ASNPs)

**Figure 1:**
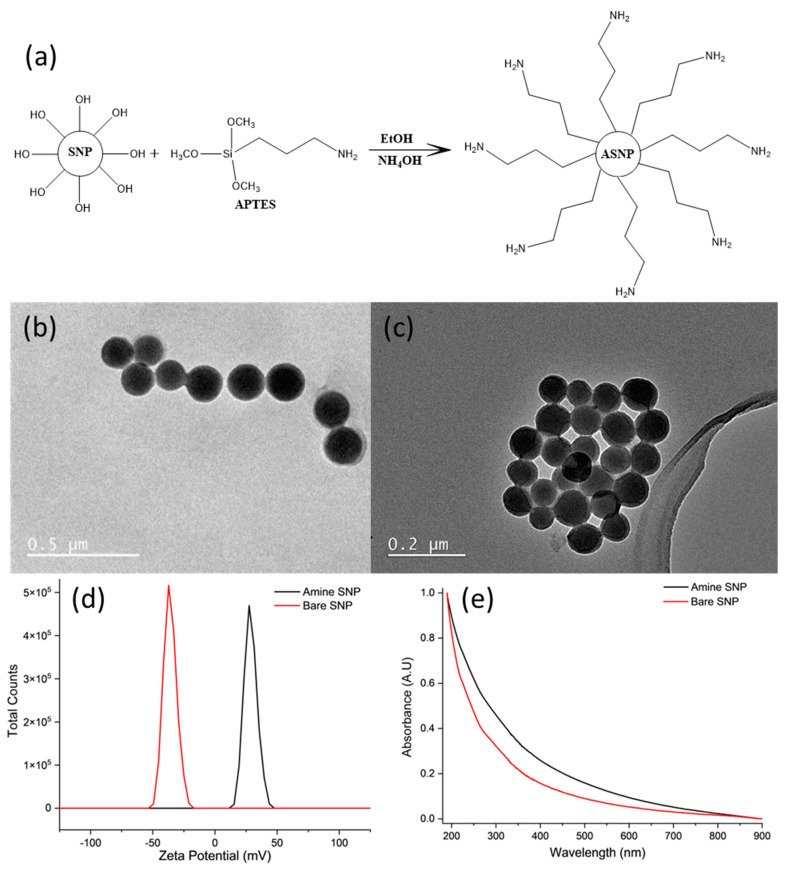
(**a**) Surface functionalization of silica nanoparticles (SNPs) by APTES to produce amine functionalised silica nanoparticles (ASNPs); (**b**) TEM image of bare silica nanoparticles (SNPs); and (**c**) amine silica nanoparticles (ASNPs); (**d**) Zeta potential of SNPs and ASNPs using 5 mg/mL per sample; and (**e**) UV-VIS of bare SNPs and ASNPs using 5 mg/mL per sample.

ASNPs with 100 nm diameter were successfully synthesized by post synthesis functionalization of SNPs using APTES; a schematic representation of this is shown in Figure 1a. The spherical shapes of bare SNPs (Figure 1b) and ASNPs (Figure 1c) were confirmed using TEM imaging, and size was determined by taking an average diameter of 100 individual nanoparticles using the processing program Image J. The average diameter of the ASNPs was 108 ± 6 nm (PDI 0.095), using direct light scattering (DLS). Functionalisation was confirmed using zeta potential (Figure 1d). Figure 1d shows bare nanoparticles (red line) had a zeta potential of −37 mV due to the deprotonated silanol groups on the surface of the SNPs, while ASNPs (black line) had a surface zeta potential of +27 mV due to the amine groups on the surface, in accordance with the literature [44]. Figure 1e shows functionalised amine SNPs (black line) resulted in a shift in absorbance compared to bare SNPs (red line), since functionalisation with amine groups increases light scattering, as confirmed in the literature [45]. However, no distinct peaks were visible due the dependence of optical properties on the size of nanoparticles. Studies have shown SNPs with diameters over 100 nm have a higher extinction efficiency, resulting in weak wavelength dependence, making it difficult to see small distinct peaks [46].

### 2.2. Polymers Grafts on ASNPs

**Figure 2:**
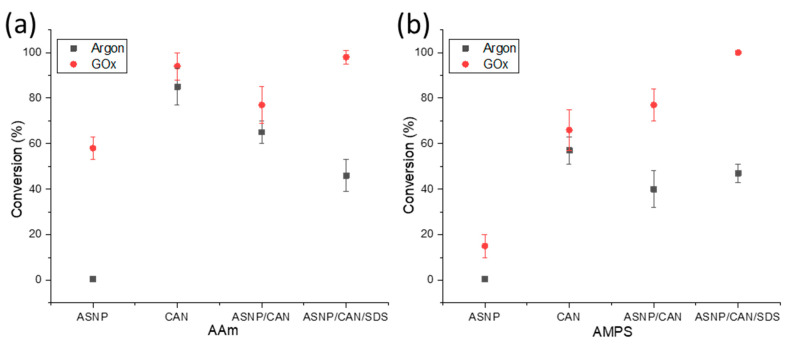
Conversion profiles of (**a**) AAm and (**b**) AMPS in combination with amine functionalised silica nanoparticles (ASNP), ASNP/CAN, and ASNP/CAN/SDS, degassed with argon (black) and GOx (red).

Both AMPS and AAm conversion profiles were investigated using samples that were degassed using either argon or GOx. The monomer and polymer peaks were calculated relative to trioxane peaks at 0 h and 24 h samples in order to calculate the final conversion profiles, using ^1^H NMR analysis as described in the supplementary information. Samples were tested using AAm and AMPS monomers with the redox initiator CAN, to compare the impact on the polymerisation through the addition of ASNPs, or SDS. Figure 2a compares the conversion percentage of AAm and AMPS, respectively, with both argon and GOx degassing. The results demonstrate that the use of GOx has a higher conversion percentage compared to argon degassing for all scenarios. GOx has been used previously as a degassing agent, and polymerisation mediator, to prevent oxygen inhibition of free radicals [14,19]. It has also been proven that GOx provides both AAm and AMPS with better reaction kinetics when compared to argon degassing [14].

An indication that H_2_O_2_, a GOx by-product, results in free radical formation which impacts the final polymer conversion can be seen by comparing argon and GOx degassed samples of AAm/ASNP. Argon degassed samples resulted in 1% conversion, whereas samples degassed with GOx had 58% conversion. This can occur during the formation of H_2_O_2_ and its subsequent in situ degradation back to water where peroxy radicals may form, potentially initiating the polymerization [47,48]. Therefore, it can be deduced that H_2_O_2_ plays a part in initiating the polymerisation to a limited extent. This was also evident for AMPS/ASNP in Figure 2b. Monomers were reacted with CAN alone, resulting in conversion of 85% with argon and 94% with GOx, for AAm. Therefore, it can be concluded CAN is an effective redox initiator for grafting AAm on the ASNPs. The results for AMPS/CAN were less notable, with only 57% conversion using argon and 66% conversion with GOx. Conversion percentage decreased with the addition of ASNPs; however, conversion reached 98% and 100% with the use of SDS for AAm and AMPS, respectively. The difference between AMPS and AAm conversion numbers under the same conditions is due to their intrinsic kinetic profiles. In a previous study, the use of amine functionalised nanoceria (ANC) as a nanoparticle-based redox initiator resulted in over 90% conversion for both AAm and AMPS, using the same nanoparticle concentration in this work [19]. ANCs, however, have the ability to use their cyclic cerium ion states to form free radicals on the tertiary amine group, compared to ASNPs that lack these intrinsic redox properties [19]. In a previous study, it was determined that the kinetic profile of AAm is more sensitive to oxygen inhibition when compared to AMPS during a photopolymerisation reaction without degassing [14]. Both AAm and AMPS had 0% conversion at 0.05 wt. % photoinitiator. The addition of GOx to AAm and AMPS with a photoinitiator concentration of 0.05 wt. % resulted in 78% and 100% conversion, respectively [14,49]. Here, higher conversion rates were achieved at the lowest tested photoinitiator concentration. This provides clear evidence on the impact GOx has on both its ability to eliminate oxygen inhibition and significantly increase the reaction kinetics of both polymers.

In the experiments, it was observed that samples degassed with argon resulted in inhomogeneous, runny, and wet gel structures. On the other hand, samples degassed with GOx showed textural consistency and more gel-like forms. These results indicate that argon alone would not be sufficient for synthesising the grafted nanocomposite hydrogels, while GOx can be used for this purpose; therefore, only GOx was used in the next part of the study.

**Figure 3:**
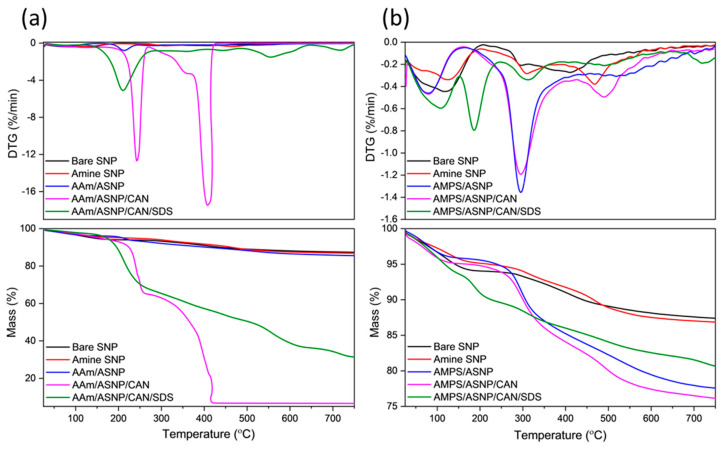
DTG and TGA profiles for (**a**) AAm and (**b**) AMPS degassed using GOx with ASNP, ASNP/CAN and ASNP/CAN/SDS, compared to Bare SNPs.

Thermal analysis was performed on dried and ground samples to investigate polymer grafting onto amine silica nanoparticles (ASNP). The mass loss profiles (TGA) in Figure 3a of AAm-based reactions showed that AAm/ASNP had a minor increase in mass loss compared to SNPs and ASNPs, indicating very low polymer grafting on the surface of the nanoparticles. This minor increase was due to the presence of a thin polymer monolayer, initiated by free radicals on the amine groups of ASNPs caused by the inert conditions created by GOx and its H_2_O_2_ by-products [14,19,50]. On the other hand, AAm/ASNP/CAN exhibited the largest mass loss, with two sharp DTG peaks at 250 °C and 420 °C (Figure 3a), indicating successful polymerisation on the surface of the ASNPs through the nature of the redox initiator, CAN, under completely inert conditions using GOx. The reaction including SDS also showed successful polymer grafting, but the presence of a distinctive DTG peak at 210 °C suggested incomplete removal of SDS from the sample. The best combination was found to be AAm/ASNP/CAN.

DTG analysis of AMPS/ASNP showed prominent polymer peaks and a mass loss of approximately 12.5%, indicating successful polymer grafts on the surface of the nanoparticles (Figure 3b). As with AAm, bare SNPs were unable to initiate PAMPS polymer formation, and minor mass loss and DTG peaks for AMPS/ASNP samples suggested low amounts of polymer grafts on the surface, likely due to the H_2_O_2_ by-products resulting in free radicals on the amine groups of ASNPs. The presence of SDS in the sample suggested successful polymer grafting, but incomplete removal of SDS from the sample even after several washes (Figure 3b). The best result was found with AMPS/ASNP/CAN, showing strong DTG polymer peaks and the largest mass loss based on polymer formation, similarly to AAm.

**Figure 4:**
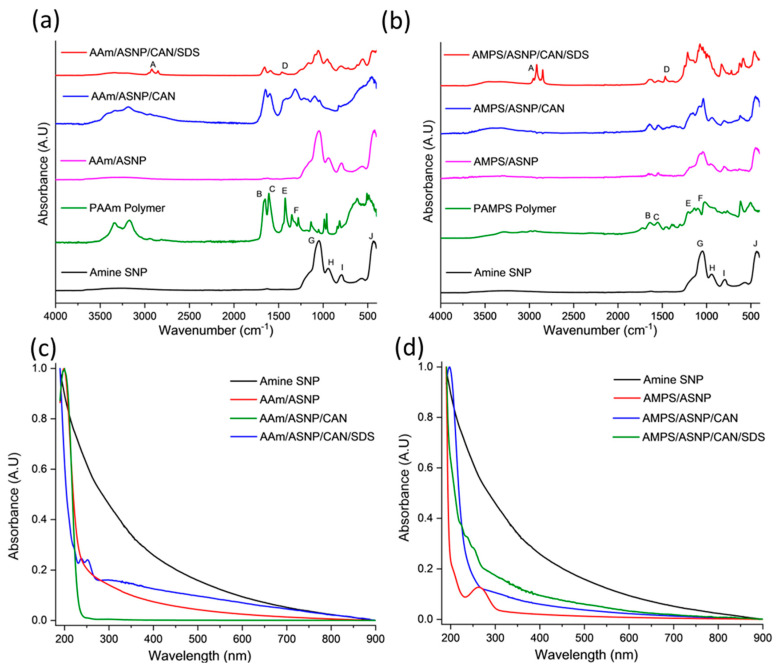
FTIR spectra post graft polymerisation of (**a**) AAm and (**b**) AMPS on the surface of amine silica nanoparticles (ASNP) compared to ASNPs, PAAm and PAMPS, degassed using GOx. Band indexing A–J shown in Supplementary Information Tables S1 and S2. UV-Vis of (**c**) AAm and (**d**) AMPS in combination with amine functionalised silica nanoparticles (ASNP), ASNP/CAN, and ASNP/CAN/SDS compared to ASNP.

FTIR analysis was conducted to confirm the surface modification of ASNPs through graft polymerisation of PAMPS and PAAm, with GOx as a degassing agent. The results of the FTIR analysis for the different combinations of PAAm with ASNP, CAN, and SDS compared to ASNP and PAAm homopolymer are shown in Figure 4a. The main bands associated with the AAm, and AMPS grafted polymers on the surface of ASNPs can be found in Tables S1 and S2 in the Supplementary Information [51,52,53,54].

The FTIR spectrum of AAm/ASNP showed no polymer bands, which is consistent with the TGA results indicating a low concentration of grafted polymer on the surface. This is likely due to the absence of an initiator to start the polymerisation between AAm/ASNP. In contrast, the FTIR spectrum of AAm/ASNP/CAN clearly showed polymer bands that reflect the PAAm spectra. The presence of bands between 1620 cm^−1^ and 1659 cm^−1^ confirms the presence of N-H and C=O bonds from PAAm, while 950 cm^−1^ and 1050 cm^−1^ correspond to Si-OH and Si-O-Si, respectively, which represent ASNPs.

The FTIR spectrum of AAm/ASNP/CAN/SDS in Figure 4b also showed these bands, albeit with less intensity, and an additional twin band between 2850 cm^−1^ and 2956 cm^−1^ that represents the C-H stretching found from residual SDS [55]. The inability to remove SDS from the system despite several wash and centrifuge cycles was confirmed by the TGA analysis. The results from the FTIR analysis and TGA suggest that the best combination for grafting AAm on ASNPs was with CAN alone, as evident from the presence of polymer bands in the FTIR spectrum.

Figure 4c shows AAm polymer grafts were successfully grafted on ASNPs, with polymer peaks visible between 190 nm and 250 nm. The AAm/ASNP spectra indicates low polymer grafting on the ASNPs due to the changes in absorbance compared to ASNPs. SDS can also be seen between 220–260 nm, due to the difficulty in removing the stabiliser from the final product. AAm/ASNP/CAN had the strongest polymer peak with a reduced ASNP peak. This suggests a thick layer of polymer was grafted on the surface of the ASNPs.

AMPS/ASNP absorbance spectra (Figure 4d) revealed that a reaction had taken place on the nanoparticles. The reduced absorbance for ASNPs in the AMPS/ASNP sample suggests the nanoparticles were covered in polymer, though the peak at 190 nm was very subtle. AMPS/ASNP/CAN spectra revealed graft polymerisation was successful. The addition of SDS also revealed successful polymerisation, with a peak at 220–260 nm suggesting SDS was still present in the sample. These results indicate that grafting PAMPS on the surface of silica nanoparticles was successful. These results provide an indication that the polymerisation technique was successful.

### 2.3. Nanocomposite Polymer Grafted Silica Nanoparticle Hydrogels (gSNP Gels)

**Figure 5:**
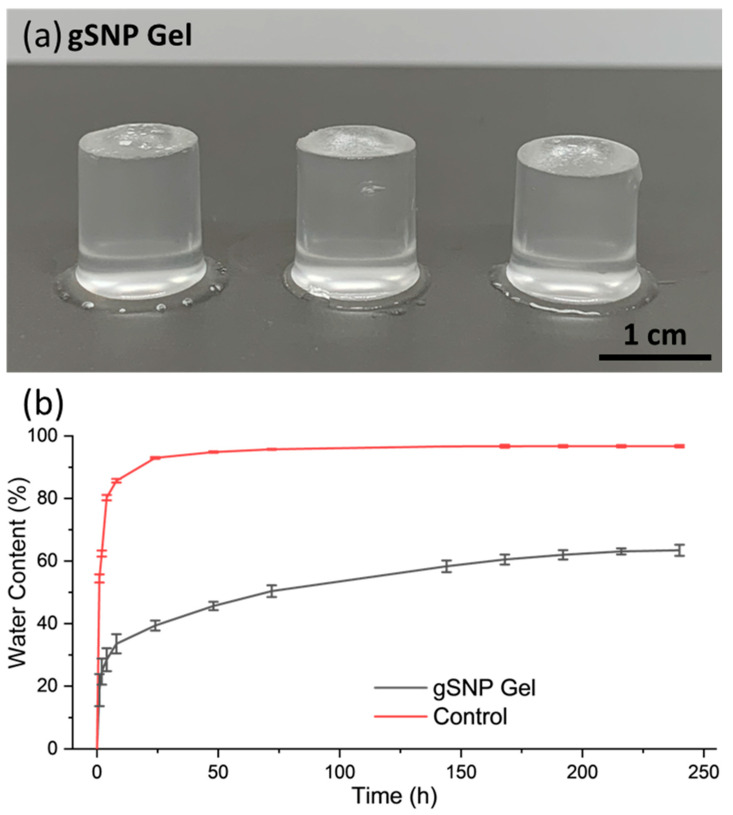
(**a**) Images of the amine silica nanoparticle hydrogels with PAMPS and PAAm polymer grafts (gSNP Gels). (**b**) Swelling profile for the amine silica nanoparticle hydrogel with PAMPS and PAAm polymer grafts (gSNP Gels), and Control (gels without ASNPs/GOx).

gSNP Gels were successfully synthesised using a two-step sequential thermal graft polymerisation technique adapted for this study (Figure 5a). PAMPS were grafted on the surface of ASNPs with CAN as a redox initiator, in the presence of GOx, to form the first network hydrogel. This was followed by soaking the first network in a monomer solution of AAm, suspended ASNPs, CAN, and GOx. The second network was formed through a secondary graft polymerisation under the same conditions. Samples were then swollen in water at room temperature until a mass plateau was achieved, and subsequently subjected to compression studies.

gSNP gels were swollen in DI-H_2_O from a dried state until a plateau in water content was reached. Figure 5b shows the swelling profile over time for the gSNP Gels, and the results are summarised in Table 1. The gSNP Gels exhibited fast water up take of approximately 19% at 1 h, followed by a more gradual and controlled trend to reach an average of 63.44 ± 1.76% at 240 h. The final swelling value was 274 ± 9.21%. The presence of ASNPs reduced the free space for water uptake due to tighter cross linking by not allowing the gels to expand as freely. In addition, ASNPs in both networks take up more space in the material as opposed to having one network cross linked via nanoparticles. Ultimately, this resulted in a slower and more controlled swelling profile containing less water, allowing for a tailorable material compared to previous DNHGs, and nanocomposite gels that often result in water content of +90% [8,10,19,56]. These values are considered in the lower range of the water content found in native articular cartilage (65–70%).

**Figure 6:**
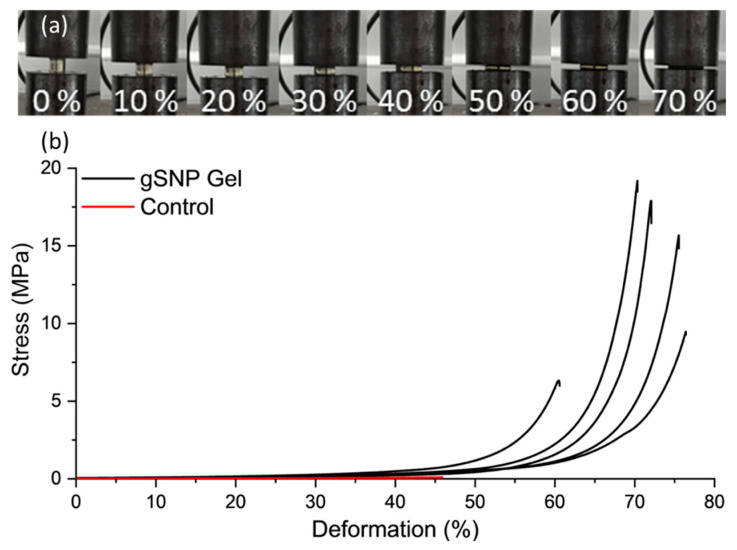
(**a**) Images of a nanocomposite PAMPS and PAAm grafted silica nanoparticle hydrogel (gSNP Gel) under compression at increasing strains. (**b**) Compression curves of the gSNP Gel, compared to a control hydrogel without ASNPs/GOx.

Figure 6 shows the compression curves for gSNP Gels, and Table 2 provides a summary of the results. An average compressive stress of 13.9 ± 5.5 MPa with fracture strain of 69.6 ± 6.4% was achieved for gSNP Gels, two orders of magnitude greater than control gels. The values for these hydrogels are comparable to the compressive strength of articular cartilage, with values ranging between 5 MPa and 20 MPa [57,58]. The compressive strength of nanocomposite hydrogels ranges from ~100 kPa to 70 MPa [12,59,60,61]. However, the results are highly dependent on testing methods and sample size during compression. Nonetheless, the improved compressive strength relative to control gels as well as other nanocomposite gels can be attributed to the covalently bonded polymer grafts on the surface of the ASNPs. The gSNP Gels sustained strains up to 75%, likely due to the internal structure of material. The polymer-ASNP composite structures are likely to interact and cross link with each other, leading to an increase in resistance to stress. This will lead to an increase in the compressive strength and allow the material to resist larger strains, as witnessed in the strain values in Figure 6. Ultimately, the gSNP Gels here show a seven fold improvement in maximum compression strength relative to nanocomposite polymer grafted ANC Gels [19]. The synthesis method used in this work can be used to tailor the mechanical properties of these hydrogels further by varying the concentration of the two polymer-ASNP grafted networks. These gSNP Gels are intended for cyclic applications such as cartilage repair or for bone tissue regeneration.

**Figure 7:**
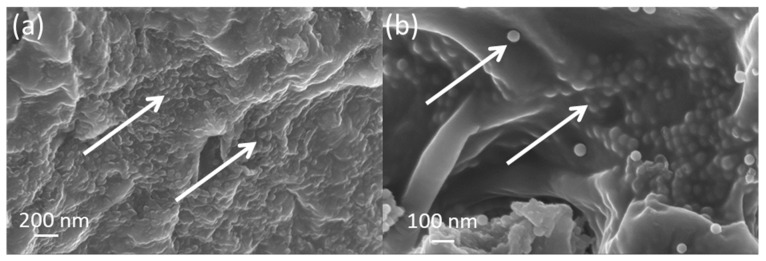
SEM images of nanocomposite PAMPS and PAAm grafted silica nanoparticle hydrogels (gSNP Gel) at varying magnifications: (**a**) 30K×, (**b**) 50K×. ASNPs are highlighted with white arrows.

Cross sections of the gSNP Gels were freeze dried and investigated under SEM, shown in Figure 7. ASNPs were well distributed across the core structure of the hydrogel with homogenous layers, as shown by the white arrows in Figure 7a,b. The arrangement of the ASNPs within the hydrogel suggests that the synthesis technique did not hinder even distribution within the final material. The polymers on the surface of the ASNPs are likely to interact and cross link with neighbouring polymer-ASNP structures, leading to a more compact structure and an increase in resistance to stress. This compact ASNP arrangement and integration into the core structure revealed by the SEM reflects the increase seen in compressive strength of the material.

## 3. Conclusions

A double network inspired nanocomposite hydrogel made of first network PAMPS and second network PAAm, both grafted on amine functionalised silica nanoparticles in the presence of GOx, was successfully synthesised in this work. Polymer conversion for both PAMPs and PAAm was best for samples containing ASNP/CAN/SDS in the presence of GOx, both achieving 100% conversion. The addition of GOx across all samples resulted in higher conversion % compared to controls degassed with argon. Ultimately, GOx proved to enhance the reaction kinetics profiles of both polymers compared to argon, resulting in more consistent gel-like polymers. GOx also showed an improvement in polymer conversion in the absence of the redox initiator CAN and allowed for open vessel synthesis due to its ability to maintain an oxygen free system. This was due to H_2_O_2_, a by-product of GOx, forming peroxy radicals that caused free radical formation on the tertiary amine group attached to the ASNPs. The grafted polymer networks were then sequentially polymerised to form a nanocomposite hydrogel with enhanced mechanical properties compared to a control without grafted ASNPs. A strong hydrogel with compressive fracture stress of 13.9 MPa at 69.6% strain and 63.4% water content was synthesised in an open vessel system with GOx. This compressive strength and water content are suitable for bone tissue engineering, as it exhibits mechanical properties comparable to soft tissues and articular cartilage. The tailorability of this hydrogel, achieved through the unique synthesis technique and the incorporation of GOx and ASNPs, opens up new possibilities for various applications in biomaterials. Future studies are needed to investigate the biological response of the nanocomposite hydrogels developed in this work. These hydrogels offer a feasible strategy to obtain tough scaffolds with enhanced mechanical properties, cell affinity, and osteoconductivity. They can also be loaded with bioactive molecules, such as growth factors or stem cells, to fabricate multifunctional hydrogels with the potential to direct bonding of the scaffold to the host bone and stimulate new bone formation.

## 4. Materials and Methods

Reagents were purchased from Sigma-Aldrich (Burlington, MA, USA) and used as received, unless stated otherwise: acrylamide (AAm; ≥99%) and 2-acrylamido-2-methyl-1-propanesulfonic acid (AMPS; 99 %); N, N’-methylenebisacrylamide (MBIS; 99%); photoinitiator 2-hydroxy-4′-(2-hydroxyethoxy)-2-methylpropiophenone (Irgacure 2959; 98%), 1, 3, 5 –trioxane (≥99%); (3-aminopropyl), ammonium hydroxide (28–30% NH_4_OH basis), tetraethyl orthosilcate (TEOS; 98%), triethoxysilane (APTES; ≥98%), deuterium oxide (D_2_O; 99.9 atom % D), cerium (IV) ammonium nitrate (CAN; ≥99.99% trace metals basis), sodium dodecyl sulphate (SDS; ≥98.5%). No additional processing and/or purifications were performed. D-glucose (G) and glucose oxidase (GOx; from aspergillus niger as a lyophilized powder) were purchased from Sigma-Aldrich (Burlington, MA, USA) and stored in phosphate buffer saline (PBS) aliquots at −20 °C when received.

Amine functionalized silica nanoparticle (ASNP) synthesis, the procedure for polymer conversion studies of argon degassing and Gox, and the characterisation techniques used in this work can be found in the supplementary information. A schematic representation of ASNP synthesis can also be found in the Supplementary Information Figure S1. TEM images of the polymer-ASNPs can be found in the Supplementary Information Figure S2 [14,47,62,63,64,65].

### 4.1. Polymer Grafted Silica Nanoparticle Hydrogels (gSNP Gels) Synthesis

The first network was formed by dispersing 150 mg of ASNPs in 5 mL of H_2_O and sonicating the mixture until it was fully dispersed. Then 4.5 g AMPS was titrated to 5.4 pH using NaOH (0.25 M), to a final concentration of 0.24 M AMPS. ASNPs were then added to the AMPS monomer solution. Then 1 wt. % MBIS and 200 nM GOx/G [14,47] were added to the monomer/ASNP solution, followed by 0.7 g CAN, and mixed for 5 min. The solution was then distributed into aliquots of 2 mL in polystyrene moulds and placed in a sonication bath set to 40 °C to ensure homogenous ASNPs dispersion in the mixture. The optimum temperature for a GOx catalysed D-glucose oxidation is 40 °C at pH 5.5, with denaturisation occurring at 60 °C [66,67]; and 40 °C being the temperature at which CAN becomes active to initiate the redox polymerisation [68]. Once the solutions have gelled, the moulds were placed in an oven at 40 °C for 24 h followed by a further 6 h at 60 °C to denature GOx.

**Figure 8:**
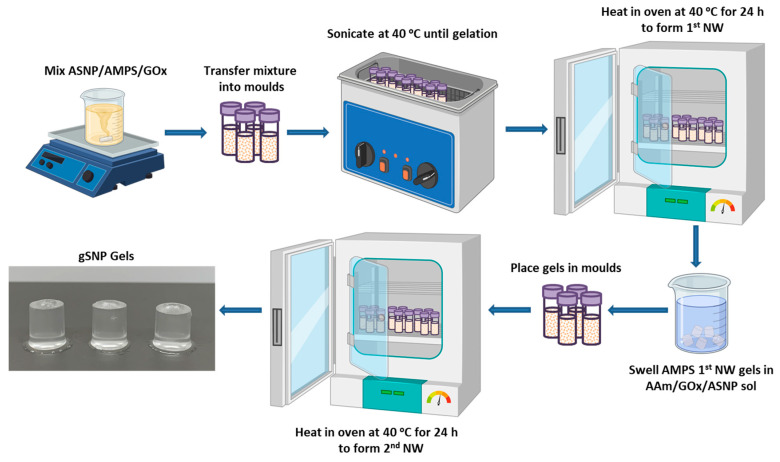
Schematic representation showing the synthesis route for polymer grafted silica nanoparticle gels (gSNP Gels).

The hydrogels were removed from the moulds and soaked in a monomer solution of 2.54 M AAm containing 150 mg ASNP, 0.1 wt. % MBIS, 0.7 g CAN, and 200 nM GOx/G. Once the hydrogels were swollen, they were placed in moulds and put into a 40 °C oven to form the second network. The final hydrogels are referred to as gSNP gels. The gSNP gels were then dried at 60 °C and, at their dry state, placed in water to swell for a week. Figure 8 shows a schematic representation of the hydrogel synthesis route described above. A comparison control hydrogel was produced using the same concentrations of AMPS (first network) and AAm (second network) along with MBIS as a crosslinker, by means of a sequential FRP process, but without the presence of ASNPs and GOx.

### 4.2. Sample Nomenclature

Each sample is named based on the chemicals used in its polymerization. For instance, if AMPS is polymerized using CAN and SDS on the surface of ASNPs, the sample will be named as AMPS/ASNP/CAN/SDS. If AAm is polymerized on ASNPs without any initiator or stabilizer, the sample will be named as AAm/ASNP.

## Figures and Tables

**Table 1:** Swelling and water content for the amine silica nanoparticle hydrogel with PAMPS and PAAm polymer grafts (GSNP Gel).

	Control Gel (No ASNPs/GOx)	gSNP Gel
Water Content (%)	96.7% ± 0.4	63.4% ± 1.8
Swelling (%)	2757% ± 157	274% ± 9

**Table 2:** Fracture compressive stress and strain values for amine silica nanoparticles hydrogels with PAMPS and PAAm polymers grafts (gSNP Gel).

	Control Gel	gSNP Gel
Compressive Fracture Stress (MPa)	0.10 ± 0.06	13.9 ± 5.5
Fracture Strain (%)	45.9 ± 2.1	69.6 ± 6.4

## Data Availability

Data available upon request from authors.

## Supplementary Materials

The following supporting information can be downloaded at: https://www.mdpi.com/article/10.3390/gels9060486/s1, Figure S1: Schematic of amine surface functinonalisation of silica nanoparticles, and graft polymerisation of AMPS and AAm on silica nanoparticles; Table S1: Summary of FTIR bands for AMPS grafted polymers on the surface of amine silica nanoparticles; Table S2: Summary of FTIR bands for AAm grafted polymers on the surface of amine silica nanoparticles. Figure S2: TEM images of polymer-ASNPs.
