# Supplementary material for: Nanocomposite Hydrogels with Polymer Grafted Silica Nanoparticles, Using Glucose Oxidase
Source: Gels. 2023 Jun 13;9(6):486. doi: 10.3390/gels9060486 (PMC10298067; doi:10.3390/gels9060486)
Supplement: Supplementary file 1 [file gels-09-00486-s001.zip › gels-2443857-supplementary.pdf]

Amine functionalized silica nanoparticle (ASNP) synthesis.

Silica nanoparticles (SNPs) were synthesized based on an optimized Stöber process to produce monodispersed SNPs[1,2]. SNPs with 100 nm diameter were synthesized by measuring out 82.33 ml EtOH and 10.30 ml (6 M) deionised water (DI-H_2O) into a beaker. Whilst the mixture was stirring, 1.11 ml (0.28 M) NH_4OH, were added to the solution followed quickly by 6.25 ml (0.28 M) TEOS as identified by Greasley et al. [2]. The solution was left to stir at 500 rpm for 24 h at room temperature. The final milky white solution was centrifuged using the Eppendorf 5430 Centrifuge and washed with EtOH three times at 7,830 rpm to remove any unreacted TEOS. The final precipitate was left to dry at 60 ^oC overnight.

The SNPs were then functionalised with amine groups[3,4] by dispersing 500 mg of dried SNPs in 10 ml EtOH and then sonicated using the CamSonix 1800T until a homogenous dispersion was achieved. The solution was then topped up with 90 ml EtOH whilst stirring. 500 µl anhydrous NH_4OH and 3 ml APTES were added to the solution and left to stir for a further 24 h. Once the reaction was complete, the solution was centrifuged and washed three times with EtOH to remove any unreacted APTES. The final precipitate was dried at 60 ^oC and stored under dry conditions. Figure S1 shows the surface functionalization chemistry of SNPs using APTES.

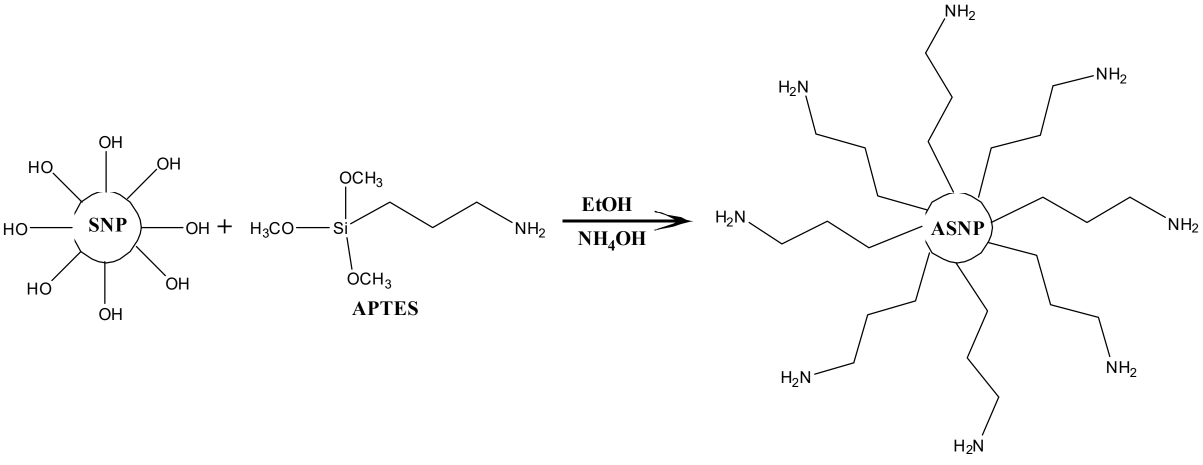

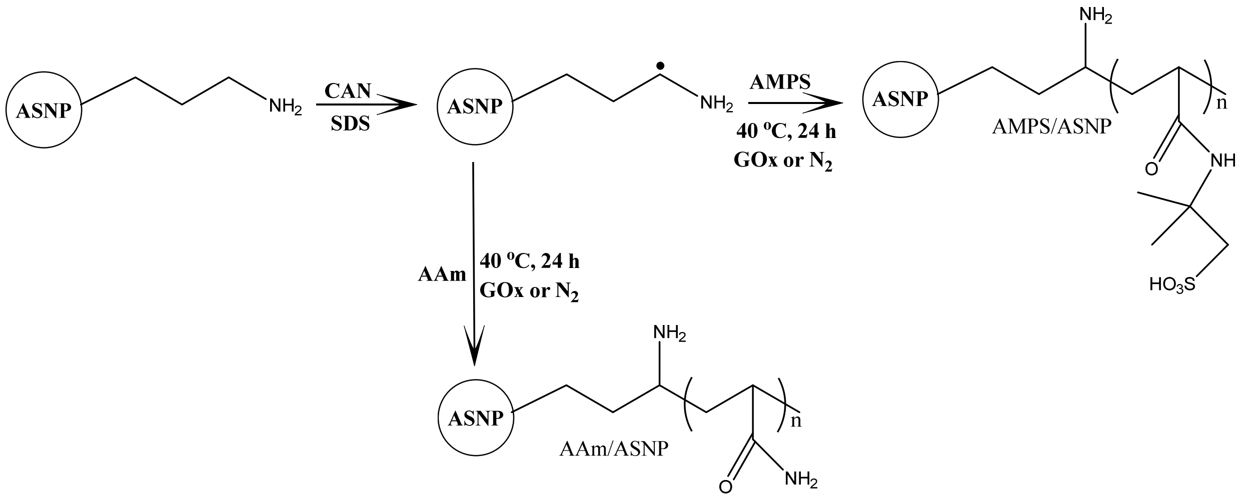

Figure S1. (Top) Surface functionalization of silica nanoparticles (SNPs) by APTES to produce amine functionalised silica nanoparticles (ASNPs). (Bottom) Grafting AMPS and AAm on the surface of amine functionalized silica nanoparticles (ASNP) via CAN or CAN/SDS, degassed using either GOx or nitrogen gas.

Polymer conversion studies using argon degassing and Gox.

Argon

150 mg ASNPs were dispersed in 5 ml of DI-H_2O and sonicated for 1 h until fully dispersed. 5 mg of trioxane and 4.5 g AMPS (0.24 M) were dissolved in 35 ml DI- H_2O in a round bottom flask until the solution was clear. Dispersed ASNPs were then added to the monomer solution and mixed for 5 minutes. A 20 µl sample was taken as a 0 h reference and topped up with 0.5 ml D_2O in a ^{1}H NMR tube to calculate polymer conversion. The solution was then degassed for 20 min with argon gas while mixing at 40 °C in a paraffin bath. The reaction was stopped at 24 h and a 20 µl sample was taken for ^{1}H NMR to calculate the final conversion. The content of the round bottom flask was centrifuged using the Eppendorf 5430 at 7,380 rpm for 15 min then washed twice and left to dry at 40 °C. The process was repeated a second time with CAN 0.7 g being added to the monomer solution, and a third time with CAN 0.7 g with 1.5 g SDS

being added to the monomer solution. The same process was repeated for AAm under the same conditions.

GOx

5 mg of trioxane and 4.5 g AMPS were dissolved in DI-H_2O and titrated to 5.4 pH using NaOH (0.25 M), then topped up to 35 ml with DI-H_2O with a final concentration of 0.24 M AMPS. 150 mg ASNPs were dispersed in 5 ml H_2O and sonicated for 1 h then added to the monomer solution. GOx/G was added at a final concentration of 200 nM, based on previous studies showing this is an optimal concentration to remove oxygen[5,6]. The solution was transferred to a round bottom flask and the process continued as described above. Repeats were carried out to include CAN, and CAN/SDS. The same process was repeated, without titration, for AAm (2.54 M).

A chemical schematic is shown in the supplementary information (Figure S1)Figure S1**Error! Reference source not found.**.

Characterisation

SNPs were diluted in ethanol, sonicated and then dropped onto 300 mesh copper TEM grids coated with holey carbon film. A JEOL FX2000 was used for imaging with tungsten filament as the electron source at an operating voltage of 200 kV and a 10 µm objective aperture. Mean diameter was calculated by measuring 100 separate SNPs.

Size analysis was also conducted using Direct light scattering (DLS). Sample volumes of 100 µl were diluted in 1.5 ml of DI-H_2O and sonicated using the CamSonix 1800T until the nanoparticles were suspended. Glass quartz cuvettes were used for analysis in the Nano ZS ZEN3600 Zetasizer, and each sample was run 3 times with 12 scans.

Samples were diluted to 3 mg in 1 ml DI-H_2O solutions and sonicated until fully dispersed for UV-VIS analysis. Samples were then loaded into the PerkinElmer Lambda 25 UV-VIS spectrometer and run against a blank sample of DI-H_2O.Swollen samples were used for mechanical analysis in compression using a Zwick Roell z2.5 machine fitted with a load cell of 10 kN and a strain rate of 1.5 mm min^{-1}. Samples tested under compression had dimensions of 10 mm x 12 mm (diameter x height).

Samples for SEM were frozen overnight at -80 °C and then freeze dried for 24 h. Samples were placed on SEM sample holders and held by carbon tape. The LEO Gemini 1525 FEG-SEM was used to image the surface morphology of freeze-dried hydrogels using 3 – 5 kV and a working distance of 5 – 10 mm.

FTIR samples were dried at 60 ^{o}C and ground to a fine powder for prior to analysis. A Nicolet iS10 Thermo Scientific FTIR was used. Samples were scanned 32 times per run with a resolution of 6 in a wavenumber region of 4000 – 400 cm^{-1}. Samples were then sputter coated for 2 minutes at 20 mA to form a 10 nm layer of chromium.

TGA was carried out for all swollen samples to study thermal the degradation of the polymer grafted silica nanoparticles relative to bare SNPs and ASNPs. The ramp rate used was 10 °C / minute under nitrogen atmosphere. The initial temperature used was room temperature (approximately 25 °C) and final temperature of 800°C. Swollen samples were dried for 5 days at 60 ^{o}C and ground to a powder. Polymeric material in the samples was expected to be burnt off by 700 ^{o}C and any remaining residues are considered to be SNPs.

Samples were swollen in DI-H_2O until a plateau in water uptake was achieved. All DNHGs exhibited a 'flowering' pattern during the swelling process in the first 24 h of swelling. This is due to the difference in hydrophilicity of the two polymer networks, resulting in different rates

of water uptake until both networks are fully saturated. Swelling (%) was calculated using Equation (S1), and water content was calculated using Equation (S2):

$$\text{Swelling (\%)} = \frac{M_{s(t)} - M_d}{M_d} \times 100\% \quad \text{Equation (S1)}$$

$$\text{Water content (\%)} = \frac{M_{s(t)} - M_d}{M_{s(t)}} \quad \text{Equation (S2)}$$

Where $M_{s(t)}$ is the swollen mass of the sample at time (t), and M_d is the initial dry mass of the sample.

Table S1. Summary of FTIR bands for AMPS grafted polymers on the surface of amine silica nanoparticles (ASNP) [7-10].

	Bond	Type	Wavenumber (cm^{-1})
			2956
A	C-H	Stretching	2918
			2850
B	C=O	Stretching, coupled with N-H bending	1652-1659
C	N-H	Primary amine	1620
D	CH2	Bending	1467
E	N-H	Bending	1238
F	R–S(=O)–R'		1040
G	Si-O-Si	Asymmetric stretching	1050
H	Si-OH	Stretching	950
I	Si-O-Si	Bending	800
J	S-O-Si	Bending	440

Table S2. Summary of FTIR bands for AAm grafted polymers on the surface of amine silica nanoparticles (ASNP) [7-10].

	Bond	Type	Wavenumber (cm^{-1})
			2956
A	C-H	Stretching	2918
			2850
B	C=O	Stretching, coupled with N-H bending	1652-1659
C	N-H	Primary amine	1620

D	CH_2	Bending	1467
E	N-H	Bending	1238
F	R–S(=O)–R'		1040
G	Si-O-Si	Asymmetric stretching	1050
H	Si-OH	Stretching	950
I	Si-O-Si	Bending	800
J	S-O-Si	Bending	440

TEM imaging was used to further examine the formation of polymer growth on the surface of ASNPs. Figure S2 (a) and (b) show the formation of PAMPS polymeric masses surrounding silica nanoparticles, indicated with white arrows. This reaffirms that the introduction of CAN is necessary to initiate the polymerisation on the surface of the nanoparticles. Samples with AAm were also examined, revealing similar polymeric masses as shown Figure S2 (c) and (d) indicated with white arrows.

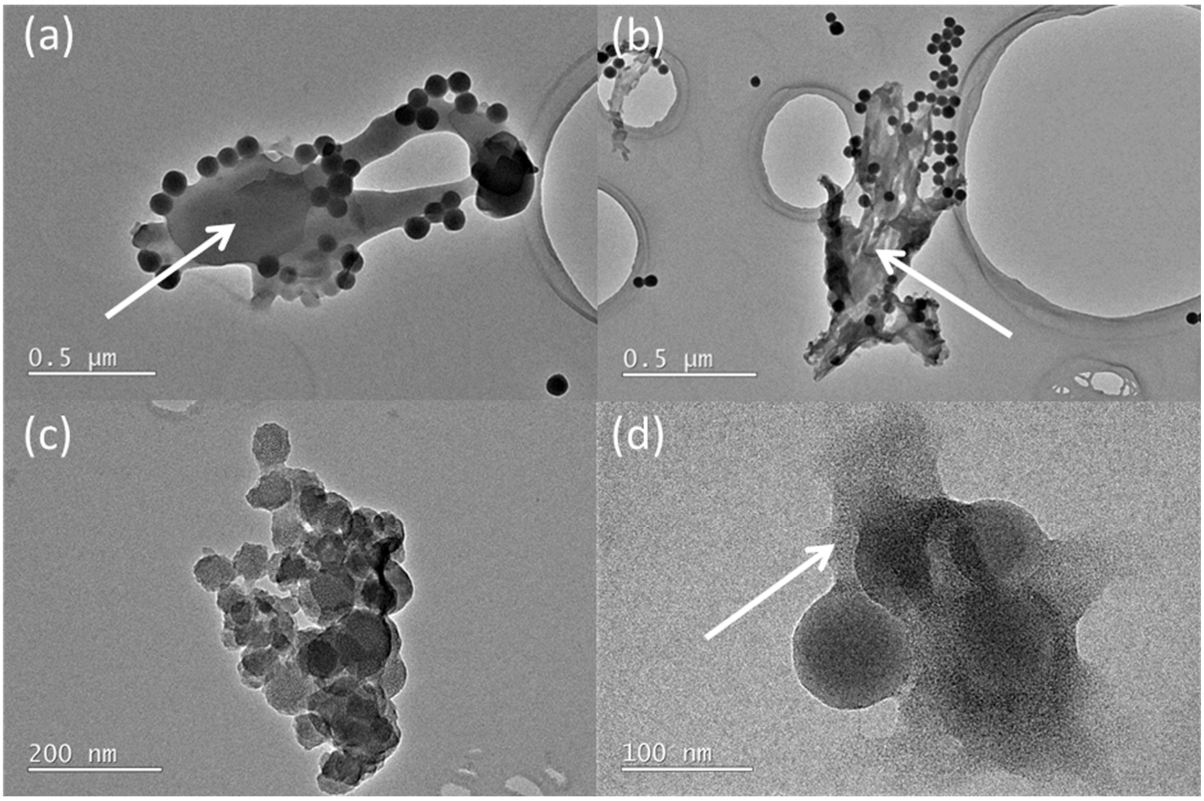

Figure S2. (a, b) TEM images of an AMPS/ amine silica nanoparticles (ASNP)/ CAN sample, showing polymeric mass surrounding ASNPs, highlighted with white arrows. (c, d) TEM images of AAm/ amine silica nanoparticles (ASNP)/ CAN sample, showing polymeric mass surrounding ASNPs, highlighted with white arrows.

References:

1. Stöber, W.; Fink, A.; Bohn, E. Controlled growth of monodisperse silica spheres in the micron size range. *Journal of Colloid and Interface Science* **1968**, *26*, 62-69, doi:http://dx.doi.org/10.1016/0021-9797(68)90272-5.
2. Greasley, S.L.; Page, S.J.; Sirovica, S.; Chen, S.; Martin, R.A.; Riveiro, A.; Hanna, J.V.; Porter, A.E.; Jones, J.R. Controlling particle size in the Stöber process and incorporation of calcium. *Journal of Colloid and Interface Science* **2016**, *469*, 213-223, doi:https://doi.org/10.1016/j.jcis.2016.01.065.
3. Rahman, I.A.; Padavettan, V. Synthesis of Silica Nanoparticles by Sol-Gel: Size-Dependent Properties, Surface Modification, and Applications in Silica-Polymer Nanocomposites—A Review. *Journal of Nanomaterials* **2012**, *2012*, 132424, doi:10.1155/2012/132424.
4. Rahman, I.A.; Jafarzadeh, M.; Sipaut, C.S. Synthesis of organo-functionalized nanosilica via a co-condensation modification using γ-aminopropyltriethoxysilane (APTES). *Ceramics International* **2009**, *35*, 1883-1888, doi:https://doi.org/10.1016/j.ceramint.2008.10.028.
5. Mohammed, A.A.; Aviles Milan, J.; Li, S.; Chung, J.J.; Stevens, M.M.; Georgiou, T.K.; Jones, J.R. Open vessel free radical photopolymerization of double network gels for biomaterial applications using glucose oxidase. *Journal of Materials Chemistry B* **2019**, *7*, 4030-4039, doi:10.1039/C9TB00658C.
6. Chapman, R.; Gormley, A.J.; Herpoldt, K.-L.; Stevens, M.M. Highly Controlled Open Vessel RAFT Polymerizations by Enzyme Degassing. *Macromolecules* **2014**, *47*, 8541-8547, doi:10.1021/ma5021209.
7. Kitamura, N.; Yasuda, K.; Ogawa, M.; Arakaki, K.; Kai, S.; Onodera, S.; Kurokawa, T.; Gong, J.P. Induction of Spontaneous Hyaline Cartilage Regeneration Using a Double-Network Gel. *The American Journal of Sports Medicine* **2011**, *39*, 1160-1169, doi:10.1177/0363546511399383.
8. Stoilova, O.; Koseva, N.; Manolova, N.; Rashkov, I. Polyelectrolyte complex between chitosan and poly(2-acryloylamido-2-methylpropanesulfonic acid). *Polymer Bulletin* **1999**, *43*, 67-73, doi:10.1007/s002890050534.
9. Pinna, A.; Malfatti, L.; Galleri, G.; Manetti, R.; Cossu, S.; Rocchitta, G.; Migheli, R.; Serra, P.A.; Innocenzi, P. Ceria nanoparticles for the treatment of Parkinson-like diseases induced by chronic manganese intoxication. *RSC Advances* **2015**, *5*, 20432-20439, doi:10.1039/C4RA16265J.
10. Ting, S.R.S.; Whitelock, J.M.; Tomic, R.; Gunawan, C.; Teoh, W.Y.; Amal, R.; Lord, M.S. Cellular uptake and activity of heparin functionalised cerium oxide nanoparticles in monocytes. *Biomaterials* **2013**, *34*, 4377-4386, doi:https://doi.org/10.1016/j.biomaterials.2013.02.042.
